# Narrowband‐Ultraviolet B Phototherapy for Psoriasis Treatment in Skin of Color: A Systematic Review and Meta‐Analysis

**DOI:** 10.1111/phpp.70051

**Published:** 2025-09-04

**Authors:** Megan Hauptman, Amjad El Othmani, Svati Pazhyanur, Mio Nakamura

**Affiliations:** ^1^ Department of Dermatology University of Miichigan Ann Arbor Michigan USA; ^2^ School of Medicine Wayne State University Detroit Michigan USA

**Keywords:** narrow band‐UVB phototherapy, psoriasis, skin of color

## Abstract

**Background:**

Narrowband‐ultraviolet B (NB‐UVB) phototherapy is an effective treatment for psoriasis in patients who have failed topical regimens or those who desire to avoid systemic treatment. Despite its regular use in non‐white individuals, NB‐UVB treatment response for psoriasis in skin of color (SOC) has not been systematically reviewed.

**Methods:**

We conducted a systematic review on the basis of the Preferred Reporting Items for Systematic Reviews and Meta‐Analyses (PRISMA) on all available studies to date assessing NB‐UVB for psoriasis treatment in skin of color (SOC) (up to 15 November 2024). The primary outcome was qualitative data on clinical outcomes of UVB (PASI 75). Random‐effects meta‐analysis was performed to assess treatment responses. Secondary outcomes of biochemical and immunologic mechanisms of NB‐UVB, NB‐UVB in combination with other treatments, and NB‐UVB compared to other forms of phototherapy were assessed.

**Results:**

Of 1283 articles initially identified, 54 were ultimately included for formal review. We identified 43 articles assessing clinical outcomes of NB‐UVB phototherapy in patients with Fitzpatrick skin type III–IV for a total of 1322 patients with chronic plaque psoriasis and 12 patients with palmoplantar psoriasis. Nine studies were included for meta‐analysis of PASI75 response; 70.5% of patients achieved PASI75, and all studies demonstrated statistically significant PASI improvement after treatment. NB‐UVB demonstrated a higher rate of complete clearance when compared to BB‐UVB but did not result in a statistically significant difference in the proportion of the patient population achieving PASI75 when compared to PUVA.

**Conclusions:**

Phototherapy is effective for the treatment of psoriasis in SOC patients and remains a valuable treatment option despite the advent of various topical, systemic, and biologic treatments for psoriasis.

## 1 Introduction

Psoriasis is a chronic inflammatory skin disease that affects an estimated 125 million people worldwide, or 2%–3% of the total population [1], though 81% of countries in the world lack information on the epidemiology of psoriasis [2]. In the United States, psoriasis affects a diverse population, including 3.7% of White individuals, 1.9% of Black individuals, 1.6% of Hispanic individuals, and 1.4% of other races/ethnicities [3].

Multiple treatment modalities are utilized for psoriasis, including topical agents, systemic therapies, biologics, and phototherapy. Narrowband‐ultraviolet B (NB‐UVB) phototherapy is a mainstay treatment for psoriasis in patients who have failed topical regimens and is especially an ideal treatment option in individuals with multiple comorbidities or those who desire to avoid systemic or biologic treatment. It is effective, with approximately 62% of patients undergoing UVB achieving at least 75% improvement in the Psoriasis Area and Severity Index (PASI75) [4].

In recent years, there have been efforts to enhance representation of non‐white individuals in dermatology research, as studies on treatment response in non‐white participants are limited. Given the regular use of NB‐UVB in psoriasis patients, information regarding its efficacy and biochemical mechanisms of action in psoriasis patients with skin of color (SOC) is invaluable. Therefore, we performed a systematic review of all available studies to date assessing NB‐UVB for psoriasis treatment in SOC.

## 2 Methods

We conducted a systematic review on the basis of the Preferred Reporting Items for Systematic Reviews and Meta‐Analyses (PRISMA) [5]. Publications (up to 15 November 2024) were searched in PubMed/MEDLINE by two independent researchers (MH, AEO). The database was searched for primary literature on UVB treatment for psoriasis in patients of SOC (Fitzpatrick skin III‐VI). The search terms “UVB AND psoriasis” was followed by countries whose population majority has Fitzpatrick Skin III‐VI (Appendix A). Additionally, UVB AND psoriasis AND Fitzpatrick III, IV, V, or VI were searched for completeness.

We screened the abstract of each article identified from the above search. The references of these articles were also searched. We then selected peer‐reviewed publications in English presenting primary research with qualitative data on clinical outcomes of UVB or data on biochemical mechanisms of action of NB‐UVB for psoriasis treatment in SOC. We conducted a meta‐analysis that included studies reporting PASI75 as an outcome. We used the percent of patients achieving PASI75 as reported in each study as the effect size. 95% Clopper‐Pearson confidence intervals (CIs) were calculated. A generalized linear mixed‐effects model and logistic transformation were used to calculate a pooled proportion of patients achieving PASI75 as well as a 95% CI. Between‐study heterogeneity was calculated using *I* ^2^ and *τ* ^2^ statistics.

## 3 Results

**FIGURE 1:**
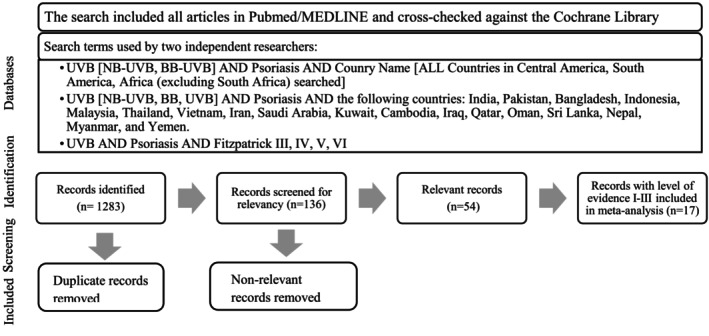
PRISMA diagram showing databases and search terms, followed by the number of articles identified and included in this review.

Following the initial search, 1283 articles were identified, and their abstracts were screened. After removing duplicates and selecting applicable articles, 54 were ultimately included for formal review. Figure 1 demonstrates the screening process. Articles were categorized into one or more of the following topics of investigation: clinical outcomes of NB‐UVB phototherapy, biochemical and immunologic mechanisms of NB‐UVB, NB‐UVB in combination with other treatments, and NB‐UVB compared to other forms of phototherapy (psoralen and UVA (PUVA) or broad band (BB)‐UVB).

### 3.1 Clinical Outcomes of NB‐UVB Phototherapy

We identified 43 articles assessing clinical outcomes of NB‐UVB phototherapy in patients with Fitzpatrick skin type III–IV. Of these, 20 were from Africa (19 Egypt, 1 Kenya) [6, 7, 8, 9, 10, 11, 12, 13, 14, 15, 16, 17, 18, 19, 20, 21, 22, 23, 24, 25, 26, 27], 21 from Asia (9 India, 1 Indonesia, 4 Thailand, 2 Vietnam, 3 Iran, 1 Saudi Arabia, and 1 South Korea) [28, 29, 30, 31, 32, 33, 34, 35, 36, 37, 38, 39, 40, 41, 42, 43, 44, 45, 46], and 1 from Europe (England) [47] for a total of 1322 patients with chronic plaque psoriasis and 12 patients with palmoplantar psoriasis. Treatment schedules varied from 2×/week for 8 weeks to 3×/week for 12 weeks. All studies demonstrated statistically significant PASI improvement after treatment.

Nine studies were included for meta‐analysis of PASI75 response, all from Asia. Of these, 3 studies included patients with Fitzpatrick skin types IV–V, 2 included patients with skin types III–IV, and 4 did not report specific skin types. Overall, 70.5% of patients achieved PASI75 (pooled prediction 0.71 [CI 0.65–0.75]) (Table 1). There was moderate heterogeneity in this subgroup (*I* ^2^ = 45%, *τ* ^2^ = 0, *p* = 0.07).

**TABLE 1:** Clinical outcomes of NB‐UVB phototherapy in patients with Fitzpatrick skin type III–IV.

Study	Proportion pasi 75	95% CI
Boonpethkaew et al. [38]	1	[0.2924; 1.0000]
Dayal et al. [35]	1	[0.8843; 1.0000]
Chauhan et al. [32]	0.8095	[0.5809; 0.9455]
Mahajan et al. [31]	0.7778	[0.5236; 0.9359]
Van et al. [43]	0.7667	[0.5772; 0.9007]
Minh et al. [42]	0.6803	[0.5898; 0.7618]
Farshchian et al. []	0.64	[0.4252; 0.8203]
Legiawati et al. [48]	0.5417	[0.3282; 0.7445]
Rattanakaemakorn et al. [41]	0.2667	[0.0779; 0.5510]
Random effects model	0.7049	[0.6496; 0.7547]

### 3.2 Biochemical and Immunologic Mechanisms of NB‐UVB

Although the exact mechanism of action of phototherapy remains unknown, multiple studies have investigated various biochemical and immunologic markers pre‐ and post‐ NB‐UVB treatment in SOC psoriasis patients (Table 2). We review below what is understood of the key processes involved in NB‐UVB treatment of psoriasis in patients with SOC:

1. Suppression of inflammatory pathways: NB‐UVB leads to the upregulation of caveolin 1, which results in decreased activation of the JAK/STAT pathway and decreased cytokine production and inflammation [7]. It increases sirtuin 1 levels and vaspin, resulting in suppression of nuclear factor kappa‐light‐chain enhancement of activated B cells (NF‐kB) and signal transducer and activator of transcription 3 (STAT3) pathways [16, 21]. Alternatively, it results in lower levels of chitinase‐3‐like protein 1 that subsequently lowers the levels of TNF‐alpha, IL‐1beta, IL‐6, and interferon gamma [13] and decreases tissue plexin‐B2 and plasmin, which subsequently lower activation of the NF‐kB signaling pathway and production of pro‐inflammatory cytokines [9, 20, 51]. Furthermore, NB‐UVB results in suppression of IL‐36 gamma (a potent inducer of IL‐23 and TNF‐alpha in macrophages), cathepsin G (activator of IL‐1 family of cytokines), and substance P (an activator of NK1R) [11, 53].
2. Modulation of immune responses: NB‐UVB increases regulatory T cells, decreases circulating and cutaneous Th1 cells, effectively reducing production of pro‐inflammatory cytokines (IFN‐gamma and TNF‐alpha) and increasing circulating and cutaneous Th2 cells (thought to have an antagonizing effect on Th1 cells) [19].
3. Reduction of oxidative stress: NB‐UVB therapy reduces oxidative stress in psoriatic lesions by modulating genes (STAT 1/3, HIF1A, IL1B, P4HB, SOD2, and MMP2) involved in redox signaling and mitochondrial quality control [52].

**TABLE 2:** Biochemical and immunologic markers pre‐ and post‐ NB‐UVB treatment in SOC psoriasis patients.

Marker	NB‐UVB regimen	Change in marker with treatment
Angiopoietin 2 [14]	3×/week for 12 weeks	Decrease
C‐reactive protein	25 sessions	Decrease
Cathepsin G [11]	3×/week for 8 weeks	Decrease
Folate [49]	2–3×/week for 30 sessions	Decrease
Homocysteine [50]	Unknown (underwent at least 30 sessions)	Decrease
IL‐36 gamma [11]	3×/week for 8 weeks	Decrease
Nuclear factor kappa B [51]	3×/week for 12 weeks (or until PASI75 reached)	Decrease
Plasmin [20]	3×/week for 8 weeks	Decrease
Psoriasis signature genes (IL36G, DEFB4A/B, S100A15, SERPINB4, KRT16, and KRT6A), IL‐17A/C, IL‐22 [38]	2–3×/week for 12 weeks	Decrease
STAT1/3, HIF1A, IL1B, P4HB, SOD2 [52]	2–3×/week for 12 weeks	Decrease
Th1 cells (circulating and skin) [19]	3×/week for 8 weeks	Decrease
Tissue plexin‐B2 [9]	3×/week for 12 weeks	Decrease
Truncated substance P [53]	3×/week for 7 weeks (20 sessions total)	Decrease
Caveolin 1 [7]	3×/week for 12 weeks	Increase
CD26/DPPIV ratio [53]	3×/week for 20 sessions	Increase
IL‐10 mRNA [25]	Unknown	Increase
MMP2, FN1 [52]	2–3×/week for 12 weeks	Increase
Sirtuin 1 [16]	2×/week for 12 weeks	Increase
Th2 cells (circulating and skin) [19]	3×/week for 8 weeks	Increase
Treg (circulating and skin) [19]	3×/week for 8 weeks	Increase
Vaspin [21]	3×/week for 8 weeks	Increase
Galectin 3 [10]	2×/week for 12 weeks	No change
Vitamin D [18]	Unknown	No change
Chitinase‐3 like protein 1^a^ [8, 13]	2×/week for 12 weeks	No change [8]/ Decrease [13]

^a^ Abu El‐Hamd [8] demonstrated no significant correlation between chitinase‐3 like protein 1 and duration of disease after treatment with NB‐UVB phototherapy; Khashaba [13] demonstrated chitinase‐3‐like protein 1 as positively correlated with IL‐17 as an inflammatory marker in psoriasis.

### 3.3 NB‐UVB in Combination With Other Treatments

NB‐UVB with mineral oil treatment demonstrated significant improvement in PASI compared to NB‐UVB treatment alone [37]. NB‐UVB with topical tacalcitol resulted in 93.3% achieving target plaque clearance by 12 weeks, with reduced mean days, NB‐UVB sessions, and cumulative dose of NB‐UVB for clearance [34]. NB‐UVB with topical tazarotene demonstrated > 98% improvement in plaque scaling, thickness, and erythema, with 100% treatment success reached in an average of 32 days (SD 5.5 days) [30]. One study investigated the use of the 308‐nm Excimer laser with 10% liquor carbonis detergens (LCD) and showed greater reduction in psoriasis scalp severity index score than the Excimer laser alone [41].

Multiple studies investigated NB‐UVB with methotrexate, all of which demonstrated a majority achieving PASI75 after 2–3×/week treatments for 12 weeks [9, 12, 31, 36, 54, 55]. One study demonstrated the combination to be efficacious in palmoplantar psoriasis as determined by the erythema, scaling, induration, and fissuring [36].

Alternatively, the combination of NB‐UVB and acitretin demonstrated no statistically significant improvement in PASI compared to NB‐UVB alone over 12 weeks of treatment, but participants who underwent combination therapy had reduced numbers of treatment sessions and lower final and cumulative doses of NB‐UVB compared to patients who received NB‐UVB alone [9, 56]. Additionally, the combination of NB‐UVB and simvastatin, NB‐UVB and tar pretreatment, and NB‐UVB and petrolatum pretreatment demonstrated no statistically significant differences in PASI compared to NB‐UVB alone after 3×/week treatments for 12 weeks [22, 44].

### 3.4 NB‐UVB Compared to Other Forms of Phototherapy

When compared to BB‐UVB, NB‐UVB demonstrated a higher rate of complete clearance (81.16% vs. 66.67% (*p* < 0.008)) of lesions after 50 sessions [6].

When compared to PUVA, NB‐UVB did not result in a statistically significant difference in the proportion of the patient population achieving PASI75 (81.8% vs. 80.9% (*n* = 43)) [32], 92% versus 80% (*n* = 50) in a second [57], and 80% versus 76.7 (*n* = 69; [43]). However, one study showed that the use of NB‐UVB resulted in a statistically significant decrease in cumulative dose (1.16 J/cm^2^ vs. 7.2 J/cm^2^ (*p* < 0.05)) and number of days to clearance (49.2 ± 20.8 vs. 65.6 ± 15.59 days (*p* < 0.05)) when compared to PUVA [30].

## 4 Discussion

Individuals with SOC face unique challenges in the diagnosis and treatment of psoriasis. Specifically, psoriasis is often misdiagnosed in SOC and presents more severely in Black patients when compared to White patients at the time of diagnosis [58, 59]. Black patients tend to experience a greater impact on quality of life than White patients, possibly because of the impact of longstanding dyspigmentation seen in SOC [60, 61].

There are many treatment modalities to consider when treating a patient with psoriasis [62]. A limited number of studies have assessed the safety and efficacy of topical therapies specifically in psoriasis patients with SOC, including calcipotriene/betamethasone dipropionate cream and foam [63, 64], halobetasol propionate 0.01% lotion [65], and halobetasol propionate and tazarotene lotion (HP/TAZ) [66]. At present, no significant differences in response to biologic treatments and safety have been reported in psoriasis patients with SOC versus White patients. However, some studies, although not statistically significant, suggest differences in treatment response among groups of individuals with SOC [62]. For example, one study demonstrated greater efficacy of secukinumab in Hispanic patients compared to non‐Hispanics. Ixekizumab had the highest score of “clear/almost clear” and PASI 75 for Asian and Latino patients. Alternatively, the highest score for “clear/almost clear” occurred in Black patients in response to brodalumab, and White patients in response to guselkumab.

Although biologics are highly efficacious, many patients around the world do not have access to systemic or biologic treatment. Some patients also have the desire to avoid systemic or biologic treatment. Furthermore, dermatology access remains a significant issue, especially among the un‐ and underinsured [67, 68], and in many developing countries. Identifying opportunities for evidence‐ and health economics‐based treatments represents an important opportunity for enhancing distributive justice in dermatologic care and can help deliver high‐value care for all patients in need. Prior studies have shown that NB‐UVB is cost‐effective, with average total home treatment costing €800 ($840) and outpatient treatment costing €752 ($789) [69]. This is a far cheaper option than biologics, with first‐year treatment ranging from $17,000 to $39,000 [70]. Thus, NB‐UVB remains an important treatment option for many patients worldwide.

Our study found that phototherapy is an effective option in SOC psoriasis patients. Of the 1334 patients of SOC included in this meta‐analysis, 70.5% achieved a PASI75 after completing NB‐UVB treatment, ranging from 2×/week for 8 weeks to 3×/week for 12 weeks. Although the biochemical mechanism by which NB‐UVB works for the treatment of psoriasis is not fully understood, it is thought to work through several molecular pathways [71]. Specifically, NB‐UVB is thought to suppress inflammatory pathways (by Th1 downregulation), reduce pro‐inflammatory cytokines (e.g., IL‐17 and IL‐22), increase peripheral blood T regulatory and Th2 cells, and decrease antigen presentation by Langerhans cells [71]. Additionally, NB‐UVB modulates the oxidative stress response and results in suppression of psoriasis signature gene expression (e.g., IL 36G, DEF4A/B, S100A15, KRT16, and KRT6A). However, most of these markers were investigated in just one or very few studies, making it difficult to assess the reproducibility or accuracy of each study's findings. Although NB‐UVB has proven to be highly efficacious, certain topical agents such as vitamin D and A derivatives, and systemic medications such as methotrexate and acitretin, can be safely added to NB‐UVB to improve efficacy or to reduce the cumulative UV dose.

There are important considerations regarding the use of phototherapy in patients with SOC, including the duration and intensity of treatment required on the basis of individual pigmentation, as well as the potential for post‐treatment hyperpigmentation, which may be undesirable to some patients. The American Academy of Dermatology and National Psoriasis Foundation guidelines specify higher starting doses and dose increments for phototherapy in skin types V–VI compared to types I–IV [72]. The estimation of the initial narrowband UVB (NB‐UVB) phototherapy dose is generally guided by skin type: 300 mJ/cm^2^ for skin types I and II, 500 mJ/cm^2^ for types III and IV, and 800 mJ/cm^2^ for types V and VI; however, the starting dose should ultimately be individualized on the basis of the patient's minimal erythema dose (MED), when applicable. MED testing is not recommended for patients with skin types V and VI because of the difficulty in detecting erythema; these patients should instead begin treatment at 800 mJ/cm^2^, with gradual increases as tolerated. At follow‐up visits, treatment response is assessed on the basis of the presence and duration of erythema, as well as subjective symptoms such as burning, stinging, pain, or itching. The dose is typically increased by 20% per session; however, before any dose escalation, patient‐reported adverse effects—such as hyperpigmentation or burns—should be carefully assessed. On the basis of clinical judgment, treatment may be withheld or maintained at the previous dose if adverse reactions are observed. The maximum recommended NB‐UVB dose is 2000 mJ/cm^2^ for skin types I and II, 3000 mJ/cm^2^ for types III and IV, and 5000 mJ/cm^2^ for types V and VI. Although these guidelines aim to assist clinicians in administering phototherapy, protocols should be tailored to each patient's history and clinical response.

This study is subject to several limitations in its evaluation of NB‐UVB for psoriasis treatment in SOC. Specifically, there is large between‐study variability because of different patient populations, study protocols, dosing methods, and follow up periods as well as sampling and measurement differences across the studies. For example, dosing varied from 2× to 3× weekly treatments, which possibly impacted efficacy rates. Another limitation is that, although the included studies centered on patients with SOC, because of limitations of search methods, some studies may have included non‐SOC individuals who resided in the countries searched. We believe this would be a very low proportion of patients. Unfortunately, none of the studies specifically included patients with Fitzpatrick VI, and few included patients with Fitzpatrick V skin. Additionally, misdiagnosis remains a significant concern, as many SOC patients with psoriasis may be misdiagnosed with atopic dermatitis or other dermatoses, leading to both underrepresentation in psoriasis studies and suboptimal, non‐targeted treatment. Furthermore, SOC patients may receive higher cumulative doses of phototherapy because of a lower risk of erythema and burning, potentially confounding assessments on treatment efficacy. Similarly, it can be challenging to clinically detect erythema in some SOC patients, which may result in underreporting of adverse effects associated with phototherapy within this population. Lastly, it is challenging to comprehensively account for confounding variables, including concomitant use of topical therapies and incidental exposure to natural light. Nevertheless, this is the first study to comprehensively investigate the effects of NB‐UVB phototherapy in psoriasis patients of SOC.

## 5 Conclusion

Phototherapy is effective for the treatment of psoriasis in SOC patients and remains a valuable treatment option despite the advent of various topical, systemic, and biologic treatments for psoriasis, especially in those who have a desire to avoid or do not have access to such treatments.

## Conflicts of Interest

The authors declare no conflicts of interest.

## Data Availability

The data that support the findings of this study are available from the corresponding author upon reasonable request.

## Appendix A Countries Included in the Literature Search

The following countries were searched: Guatemala, Nicaragua, Costa Rica, Honduras, El Salvador, Panama, Brazil, Colombia, Argentina, Peru, Venezuela, Chile, Ecuador, Bolivia, Paraguay, Uruguay, Guyana, Suriname, Nigeria, Ethiopia, Egypt, Democratic Republic of Congo, Tanzania, Kenya, Sudan, Uganda, Algeria, Morocco, Angola, Mozambique, Ghana, Madagascar, Cameroon, Niger, Mali, Burkina Faso, Malawi, Zambia, Chad, Somalia, Senegal, Zimbabwe, Guinea, Benin, Rwanda, Burundi, Tunisia, South Sudan, Togo, Sierra Leone, Libya, Liberia, Central African Republic, Mauritania, Eritrea, Namibia, Gambia, Gabon, Botswana, Lesotho, Guinea‐Bissau, Mauritius, India, Pakistan, Bangladesh, Indonesia, Malaysia, Thailand, Vietnam, Iran, Saudi Arabia, Kuwait, Cambodia, Iraq, Qatar, Oman, Sri Lanka, Nepal, Myanmar, and Yemen.
